# Resuscitating athletes

**DOI:** 10.1007/s12471-018-1096-2

**Published:** 2018-03-08

**Authors:** P. Calle

**Affiliations:** Department of Emergency Medicine, General Hospital Maria Middelares Ghent and University Ghent, Ghent, Belgium

To the Editor,

In the editor’s comment in the November issue, colleagues Panhuyzen-Goedkoop and Piek raised questions about the appropriateness of the 2015 guidelines of the European Resuscitation Council (ERC) for resuscitation attempts in athletes [1, 2]. I agree on the very high percentage of ventricular tachycardia/fibrillation (VT/VF) in athletes suddenly dying on the pitch, on a potentially important role of the athlete’s high adrenergic state, volume depletion and/or electrolyte disturbance, and on the importance of chest compressions, 112 alert and automated external defibrillator (AED) use by bystanders. I also agree that in the first minutes after the collapse caused by VT/VF, efforts for rescue ventilation are of little or even no value. Moreover, the delivery of rescue breaths needs regular training. Consequently, the ERC recommends chest compression-only cardiopulmonary resuscitation (CPR) if the bystander is untrained or unable to do rescue breaths [2, p. 87]. For trained rescuers, however, the ERC still endorses the need for rescue breaths in all cardiac arrest cases [2, p. 90]. This view should be supported until it is proven that chest compression-only CPR is beneficial under all circumstances and/or it is shown that bystanders are able to identify cases assumed not to benefit from rescue breaths on the basis of age, medical history, precipitating factors, interval between collapse and the resuscitation attempt, on-site availability of an AED, presumed response time of the emergency medical services, etcetera. In my mind, the adage ‘Keep it simple’ remains crucial, until we know that, for example, the referee trained in CPR is able to choose the most appropriate approach in a collapsed soccer player, as well as in a spectator found unresponsive in the toilets of the stadium, his neighbour with an opiate overdose, an old lady collapsing after a period of shortness of breath and a child with apnoea after long lasting seizures.

A second issue is the figures mentioned in the editor’s comment: ‘The annual incidences of sudden cardiac death (SCD; 3–10.7/100,000) and VT/VF (84.0/100,000) in the overall population are reported to be substantially higher.’[1, lines 9 to 11]. As VT/VF cases are a subgroup of the SCD patients, there is obviously a mistake when the annual incidence is (much) higher for VT/VF cases than for SCD patients. In this respect, we refer to data published by the ERC: about 55 to 113 sudden cardiac arrest cases per 100,000 inhabitants a year with a proportion of VF victims as high as 76% [2, p. 82].
